# High Burden of Human Papillomavirus (HPV) Infection among Young Women in KwaZulu-Natal, South Africa

**DOI:** 10.1371/journal.pone.0146603

**Published:** 2016-01-19

**Authors:** Sumayyah Ebrahim, Xolani K. Mndende, Ayesha B. M. Kharsany, Zizipho Z. A. Mbulawa, Vivek Naranbhai, Janet Frohlich, Lise Werner, Natasha Samsunder, Quarraisha Abdool Karim, Anna-Lise Williamson

**Affiliations:** 1 Department of Surgery, Nelson R. Mandela School of Medicine, University of KwaZulu-Natal, Durban, South Africa; 2 Division of Medical Virology and Institute of Infectious Disease and Molecular Medicine, University of Cape Town, Cape Town, South Africa; 3 Centre for the AIDS Programme of Research in South Africa, Nelson R Mandela School of Medicine, University of KwaZulu-Natal, Durban, South Africa; 4 Center for HIV and STIs, National Institute for Communicable Disease, National Health Laboratory Service, Groote Schuur Hospital, Observatory, Cape Town, South Africa; 5 Nuffield Department of Medicine, University of Oxford, Oxford, United Kingdom; 6 Department of Epidemiology, Mailman School of Public Health, Columbia University, New York, United States of America; 7 National Health Laboratory Service, Groote Schuur Hospital, Observatory, Cape Town, South Africa; Penn State University School of Medicine, UNITED STATES

## Abstract

**Objectives:**

HPV infection causes cervical cancer, yet information on prevalence and risk factors for HPV in Africa remain sparse. This study describes the prevalence of HPV genotypes and risk factors associated with HPV among young women ≤ 30 years of age in KwaZulu-Natal (KZN), South Africa.

**Methods:**

Cervicovaginal lavage samples were tested for HPV genotypes in 224 women enrolled in a prospective cohort study. Clinical, behavioural and demographic data were collected. We measured prevalence of HPV genotypes and using logistic regression, examined for factors associated with HPV.

**Results:**

Median age of participants was 21 years [interquartile range (IQR):18–23]. The overall prevalence of HPV was 76.3% (171/224) with multiple and single genotypes prevalent in 56.3% and 20.1% of women respectively. Proportion of women with high-risk genotypes (16, 18, 31, 33, 35, 39, 45, 51, 52, 56 and 58) was 54.5%. Women not living with their partner [adjusted odds ratio (aOR)] = 3.42 95% CI1.22–9.60; p = 0.019), was significantly associated with HPV infection and high-risk HPV genotype infection.

**Conclusion:**

The high burden of HPV and associated risk behaviours highlight the need to intensify behavioural interventions to prevent HPV acquisition in young women. The large scale delivery of HPV vaccine should be prioritised to prevent HPV acquisition and reduce HPV-related morbidity.

## Introduction

Human papillomavirus (HPV) is one of the most prevalent sexually transmitted viruses [1, 2]. More than 100 types of HPV have been identified, and of these about 40 types are known to infect the anogenital tract. Eighteen types; in particular, HPV 16 and HPV 18, described as high-risk/oncogenic types, are associated with the development of cervical cancer. The low-risk/non-oncogenic HPV types (mainly types 6 and 11), result in genital warts [2]. The majority of cervical HPV infections are cleared or suppressed by cell-mediated immunity within 1–2 years of exposure [3]. Persistent cervical HPV infection with high- risk/oncogenic types is associated with an increased risk of cervical intraepithelial neoplasia (CIN) and eventual malignancy [3]. Additionally, HPV is associated with non-cervical cancers such as vulval, vaginal, anal and oropharyngeal cancers [4].

Globally, the prevalence of HPV among women with normal cytology is around 12%, whilst in Sub-Saharan Africa the rates are much higher at around 24%, ranging from 17.4% to 33.6% [5]. Young women <25 years of age remain disproportionately affected with HPV prevalence of 19.2% globally, reaching a high of 43.9% in Africa in this age group [5]. Amongst women with cervical pathology, prevalence increases in proportion to the severity of lesions and reaches around 99% in women with cervical cancer, contributing to significant morbidity and mortality [1, 4]. Although risk factors for HPV acquisition have been difficult to assess these include risky sexual behaviours, particularly early sexual debut, multiple sex partners, lack of condom use, early pregnancy and hormonal contraceptive (HC) use [6]. Thus, primary prevention through education and promotion of safe sexual practices remains key to reducing HPV acquisition.

The recently developed HPV vaccines offer hope in reducing HPV acquisition, a necessary cause in the pathogenesis of cervical cancer. Two types of vaccines are currently available in South Africa: a bivalent vaccine targeting genotypes 16 and 18 and a quadrivalent vaccine targeting genotypes 6/11/16/18 [7]. The randomized controlled trials as well as a systematic review and meta-analysis, have demonstrated the efficacy of HPV vaccines for the primary prevention of HPV-related cervical malignancies and anogenital warts [8, 9]. The target population for vaccination includes young girls before the average age of sexual debut [10]. This age is determined by country-specific data on the mean age of sexual debut for girls, and includes in most instances girls <15 years old [10]. In South Africa, girls aged 9–12 years have been prioritised to receive the HPV vaccine [7] and recent data suggests (N = 963), that the overall uptake of the quadrivalent vaccine was high and it was 99.7%, 97.9% and 97.8% for the first, second and third doses respectively; with no adverse events attributable to the HPV vaccine [7].

In this study we determined the prevalence of HPV genotypes and explored the socio-demographic and behavioural factors associated with prevalent HPV among young women in KZN, South Africa.

## Methods

### Study Population and Sample Collection

This study was an ancillary study nested within a larger HIV seroincidence study conducted by the Centre for the AIDS Programme of Research in South Africa (CAPRISA) in rural and urban KZN from March 2004 to May 2007 [11]. Briefly, following informed consent, sexually active HIV-negative women aged 14–30 years attending public sector primary health care clinics for family planning services and management of sexually transmitted infections were enrolled. Nurse-administered interviews with structured questionnaires were used to collect demographic, clinical, reproductive and sexual behavioural data at baseline and at each monthly follow-up visit. At enrolment, and at each quarterly and at the study exit visit all women underwent physical and clinical examinations. Blood and cervicovaginal lavage (CVL) samples were collected for study related primary and secondary endpoints [11]. Of the 594 women enrolled, we tested CVL samples from 224 women for HPV genotypes at one time point prior to HIV seroconversion. Of the 224 women, 110 had results available for more than one time point. Participants were evaluated as HPV positive or negative depending on results of CVL genotype testing. Regarding evaluation of HPV types, all types that were detected per participant were enumerated irrespective of the time points they were obtained at. High-risk genotypes [1] were 16, 18, 31, 33, 35, 39, 45, 51, 52, 56 and 58. The first measured HPV typing data was at a median of 6.4 months (IQR 5.6–17.9) post-enrolment. The baseline characteristics of the 370 women not included for HPV testing did not differ significantly from those of the remaining women [11]. Participant identifying information was removed prior to testing and analysis. The Institutional Review Board (IRB) at Columbia University, New York approved the nested study (Reference number IRB-AAAM1102) based on study participants providing informed consent in the parent study which had been approved by the University of KwaZulu-Natal Biomedical Research Ethics Committee (Reference number E197/03). This study was also approved by the Research Ethics Committee of the University of Cape Town (Reference number 459/2011).

### Laboratory Testing

The Roche Linear Array HPV Genotyping Test (Roche Diagnostics, Mannheim, Germany) was used for the detection of 37 high- and low-risk HPV genotypes in specimens. DNA from cervico-vaginal lavage (CVL) cell pellet was extracted for HPV typing using the Magna Pure Compact Nucleic Acid Isolation Kit I (Roche Diagnostics, Mannheim, Germany). This kit was used in conjunction with the MagNA Pure Compact Instrument (Roche Diagnostics, Mannheim, Germany) employing an automated extraction procedure performed according to the manufacturer’s instruction. Samples were tested blinded of other demographic, behavioural and HIV infection status data.

### Statistical Analyses

Baseline variables were summarized as percentages for categorical variables, and mean or median for continuous variables [11]. Although a prospective cohort study was conducted, HPV genotyping data were available for 51% (114/224) of participants at only one time-point, thus logistic regression was performed to evaluate the association between baseline variables and prevalent HPV. Unordered polytomous regression techniques were used to test the associations between the baseline variables and HPV risk type (high-risk or low-risk HPV). Each baseline characteristic variable was adjusted for against the other variables in the model. Data were analysed with SAS statistical package version 9.3 (SAS Institute, Inc., Cary).

## Results

### Demographic characteristics

All 224 enrolled women were Black African with a median age of 21 years (interquartile range [IQR]: 18–23 years). Just over half (n = 129; 57.6%) of the women were from the rural area. Majority (n = 204; 91.5%) of the women were in a relationship and reported having had one sex partner (n = 203; 91.0%). Whilst a third (n = 70; 31.7%) reported use of an injectable HC, about a quarter (n = 60; 27.1%) reported using depot medroxyprogesterone acetate (DMPA) or norethisterone enantate (Nuristerate) (n = 10; 4.5%). Less than half of the women (n = 104; 47.1%) reported condom use (Table 1).

**Table 1:** Baseline characteristics associated with prevalent HPV infection in young women in KwaZulu-Natal, South Africa (N = 224).

				Unadjusted analysis		Adjusted analysis	
Baseline Characteristic		Distribution % (n)	HPV infection % (n)	Odds Ratio (95% CI)	p-value	Odds Ratio (95% CI)	p-value
**Age group (years)**							
	<18	16.5% (37)	70.3% (26)	1.0		1.0	
	≥18	83.5% (187)	77.5% (145)	1.46 (0.67–3.20)	0.344	1.28 (0.50–3.25)	0.607
**Site**							
	Rural	57.6% (129)	70.5% (91)	1.0		1.0	
	Urban	42.4% (95)	84.2% (80)	2.23 (1.14–4.35)	0.019	1.87 (0.81–4.33)	0.144
**Relationship status**^a^*							
	Single/no partner	8.5% (19)	63.2% (12)	1.0		1.0	
	With partner	91.5% (204)	77.9% (159)	2.06 (0.77–5.54)	0.152	2.08 (0.68–6.32)	0.198
**Partner has other partners**							
	No	22.3% (50)	70.0% (35)	1.0		1.0	
	Yes	25.5% (57)	75.4% (43)	1.32 (0.56–3.09)	0.528	1.12 (0.44–2.84)	0.807
	Don’t Know	52.2% (117)	79.5% (93)	1.66 (0.78–3.53)	0.187	1.46 (0.64–3.31)	0.364
**Living with partner**							
	Yes	10.7% (24)	62.5% (15)	1.0		**1.0**	
	No	89.3% (200)	78.0% (156)	2.12 (0.87–5.19)	0.100	**3.42 (1.22–9.60)**	**0.019**
**Number of sexual partners in the last 3 months**^a^							
	1 partner	91.0% (203)	74.9% (152)	1.0		1.0	
	≥2 partners	9.0% (20)	90.0% (18)	3.02(0.68–13.46)	0.147	2.00 (0.41–9.89)	0.393
**Vaginal insertion practices**							
	No	67.9% (152)	72.4% (110)	1.0		1.0	
	Yes	32.1% (72)	84.7% (61)	2.12 (1.02–4.41)	0.045	1.98 (0.88–4.48)	0.099
**Injectable contraceptive use**^b^							
	No	68.3% (151)	78.8% (119)	1.0		1.0	
	Yes	31.7% (70)	71.4% (50)	0.67 (0.35–1.29)	0.231	0.79 (0.438–1.60)	0.482
**Injectable contraceptive type**^b^							
	None	68.3% (151)	78.8% (119)	1.0			
	Nuristerate^c^	4.6% (10)	80.0% (8)	1.08 (0.22–5.32)	0.929		
	DMPA^d^	27.1% (60)	69.5% (41)	0.61 (0.31–1.21)	0.156		
**Condom use**^b^							
	Yes	47.1% (104)	79.8% (83)	1.0		1.0	
	No	52.9% (117)	73.5% (86)	0.70 (0.37–1.32)	0.271	0.68 (0.34–1.37)	0.283
**Abnormal pelvic exam**							
	No	58.0% (130)	73.1% (95)	1.0		1.0	
	Yes	42.0% (94)	80.9% (76)	1.56 (0.82–2.96)	0.178	1.43 (0.71–2.89)	0.317

Data missing for

^a^ one, and

^b^ three women

^c^ norethisterone enantate

^d^ depot medroxyprogesterone acetate

* With partner category includes married and unmarried women

### HPV prevalence and genotype distribution

HPV prevalence was 76.3% (171/224) [95% confidence interval (CI) 70.8%-81.9%]. The distribution of the genotypes is shown in S1 Fig. Multiple concurrent and single HPV genotypes were prevalent in 56.3% (126/224) and 20.1% (45/224) of women respectively. The distribution of multiple concurrent infections (Range: 2–12 HPV genotypes) is shown in S2 Fig. Among women under 18 years of age, 54.1% (20/37) had multiple concurrent HPV genotypes detected, whilst prevalence of multiple concurrent infections among women ≥18 years old was 77.4% (106/137). High-risk genotypes (1) of 16, 18, 31, 33, 35, 39, 45, 51, 52, 56 and 58 were present in 78.6% (99/126) of women with multiple concurrent and 51.1% (23/45) of women with single genotype infections. Overall the proportion of women with these high-risk genotypes was 54.5% (122/224). Among women with HPV infection, prevalence of high-risk genotypes was 71.3% (122/171). Regarding the number of participants infected with high-risk genotypes; the proportion infected with the vaccine genotypes (16±18) was 21% (47/224). In our sample, we did not find that any combinations of HPV types that were more common together.

### Risk factors for HPV

HPV was significantly associated in women who were not living with their partner (adjusted odds ratio (aOR) = 3.42; 95% CI 1.22–9.60; p = 0.019) (Table 1). Results did not reach statistical significance for the other demographic/behavioural characteristics described next. The odds for HPV were higher in women from an urban compared to a rural setting (aOR = 1.87; 95% CI 0.81–4.33; p = 0.144) and in those practicing intra-vaginal substance use of water with salt, vinegar, soap or douching with antiseptics containing parachlorometaxylenol or povidone iodine products or traditional herbs for personal hygiene before or after sex (aOR = 1.98; 95% CI 0.88–4.49; p = 0.099). Among women having more than one partner in the last 3 months, the odds of HPV was higher (aOR = 2.00; 95% CI 0.41–9.89; p = 0.393). These findings were similar among women with a partner (either married or not) (aOR = 2.08; 95% CI 0.68–6.32; p = 0.198). Neither injectable contraception use nor condom use were associated with prevalent HPV.

Among women not living with their partner, 56% (112/200) had high-risk HPV infection. These women also had higher odds of having high-risk HPV infection compared to being HPV negative (aOR = 4.08; 95% CI 1.34–12.41; p = 0.013). Similarly, women who practiced intra-vaginal substance use had 2-fold higher odds of high-risk HPV infection (aOR = 2.33; 95% CI 1.01–5.39; p = 0.049). The distribution of high-risk HPV infection in these women was 66.7% (48/72). There were no observed associations between baseline risk factors and particular genotypes.

## Discussion

The overall HPV prevalence of 76.3% in our study is almost 3–4 fold higher than reported estimates [12], demonstrating the high burden amongst both urban and rural women. Importantly this is the first study in South Africa that determined the prevalence of HPV in a rural setting (70.5%).

The prevalence of high-risk genotypes in this study was 54.5%. In a recent study of 1,472 women from the Gauteng province, the prevalence of HPV DNA was 74.6% with high- risk HPV DNA in 54.3% [12]; similar to the 56.3% in this study. A large cohort study describing the prevalence and distribution of HPV genotypes among women in Cape Town, South Africa, showed an overall prevalence of HPV DNA of 25.4% (2,389/9,429). One or more high-risk HPV genotypes were present in 78.5% (1,848/2,354) of women, which is considerably higher than that reported in our study [13]. This may be due to the fact that this study looked at the distribution of HPV genotypes among both HIV-positive and HIV-negative women.

Women 18 years and older had a higher likelihood of multiple HPV genotype infection in this study. This may be due to a biological mechanism by which the acquisition of one genotype of HPV does, in some way, facilitate acquisition of another genotype [14]. Also, sexual behaviour (frequency of intercourse, number of sex partners) or sex partner characteristics (number of HPV types in the partner/s) are important risk factors for multiple HPV genotype infection [14].There is no consensus in the literature regarding the importance of multiple concurrent infections. It is well established that increased duration of infection (persistence) with high risk HPV genotypes is associated with a concomitant increased risk of cervical dysplasia [15]. Furthermore, multiple concurrent infections may be associated with HPV persistence, hence an increased risk of cervical disease compared to single infection [16]; however this has not been consistently demonstrated [17, 18].

Epidemiological data on HPV prevalence and type distribution is crucial to evaluate the future impact of HPV vaccines. In this study, the number of women infected with vaccine high-risk genotypes was modest at 21% and is in keeping with findings from a cohort of sexually active HIV-uninfected South African adolescents (15.4%) [15]. Some authors have expressed concern that currently available HPV vaccines, even with wide coverage, may not prevent infection with the majority of high-risk HPV genotypes [15]. However, studies have shown that both the bivalent and quadrivalent vaccines show evidence of cross-protection against non-vaccine high-risk HPV genotypes [predominantly HPV 31, 33, 35 and 45] [8, 19]. This is due to the high homology of some HPV genotypes with vaccine genotypes: HPV 31/35 are closely related to HPV 16; whilst HPV 45 is more closely related to HPV 18 [19]. A nonavalent vaccine, containing virus-like-particles (VLPs) of 9 high-risk HPV genotypes (6, 11, 16, 18, 31, 33, 45, 52, and 58) currently in phase III trials, may provide broader protection against the HPV genotypes associated with cervical cancer [20].

In our study we found that HPV prevalence was high both among women <18 years (70.3%) and ≥18 years (77.5%) old. These results are higher compared to global and local trends [5, 21] and explain that most HPV infections occur soon after initiation of sexual activity, highlighting the burden of HPV at a very young age and the potential future risk of invasive cervical disease, as well as the importance of targeting young girls aged 9–12 years (in our setting) for HPV vaccination [7]. It is also important to mention that in South Africa, HIV prevalence amongst young women compared to young men is at least 5-fold higher and young women in the age group 15 to 24 years remain the worst affected [22]. Thus, over time, women receiving antiretroviral therapy will survive longer making it a priority to screen for cervical cancer in this population due to their high burden of HPV infection.

Women reported to be with a partner yet not living with their partners, were at a 3.5 times greater risk of HPV infection, and had a similar risk of having high-risk HPV infection; suggesting that unmeasured sexual behaviour and sex partner characteristics (frequency of intercourse, cumulative number of sex partners, number of previous/current partners of sex partners or number of HPV genotypes in the sex partner/s) were important risk factors [14]. Thus larger studies evaluating these sexual behaviour correlates are needed to better explain this association; being cognisant of cultural marriage practices and issues such as reporting biases of sensitive behaviours in face-to-face interviews.

Regarding the role of HC use, HPV acquisition and subsequent development of cervical cancer; findings have been inconsistent as reported in the review by Morrison *et al* [23] where an early study showed an increased risk [24], whilst a later study showed a non-significant protective effect [25]. Another study showed no association between HC use and high-risk HPV infection or on high-grade CIN and disease progression [26]. In this study we did not find an association with HC use and HPV, though genital tract alteration is known to be associated with HC use having greater likelihood of cervical ectopy and thinning of the cervico-vaginal epithelium predisposing to HPV acquisition [23]. Furthermore, HC use is linked to sexual risk behaviours, thus sexual behaviour correlates, together with better measures of HC use, must be taken into account in order to determine the true effect of HC use on HPV acquisition.

Intravaginal practices may be associated with risk of HPV infection due to alterations in the vaginal pH, microflora, or cervical mucous [27]. In this study, vaginal insertion practices were significantly associated with high-risk HPV infection. These findings are in keeping with a study among adolescent females in the United States which showed that vaginal douching during the last 90 days was associated with HPV infection (OR = 2.1, 95% CI 1.2–3.6) [27]. However, findings from a South African study did not show increased risk of HPV infection among participants who reported vaginal insertion practices [28]. Conflicting results in the literature may be due to inherent biases of self-reported sexual behaviour correlates, differing practices and types of products used.

There are several limitations in our study. It is a small sample with limited power. We did not find an association with behavioural measures and condom use and prevalent HPV; reliable measurement of behaviours including consistent condom use remains problematic since face-to-face interviews were conducted leading to reporting bias due to social desirability (perhaps an over reporting of condom use) that could attenuate any true analysis of the effect of condoms [29]. The effectiveness of condom use in reducing HPV acquisition has not been proven, but the trend is towards a protective effect [29, 30]. However a paradoxical effect has also been reported; that is condom use is associated with an increased risk of HPV acquisition probably due to the fact that women may be more likely to use a condom if they feel they are at high risk of sexually transmitted infection (STI) transmission from a partner [31]. Furthermore, the small sample size may have precluded any meaningful interpretation of the association between risk factors and particular genotypes; as well as the detection of any combinations of HPV types that were more common together.

Our small sample size and the target population of Black African women aged 14–30 years limits generalizability to all women in South Africa, including women outside sub-Saharan Africa. Nevertheless, our population represented young sexually active women at risk of acquiring infections that are sexually transmitted. Whilst we used a molecular method for genotype identification, testing was carried out on stored samples and it is possible that there could be over time, some deterioration of sample quality with HPV positive samples misclassified as false-negative. Despite this misclassification HPV prevalence was as high and similar to reports from this region.

## Conclusion

The prevalence of HPV among women <18 years of age was extraordinarily high and it is therefore imperative that there should be determined efforts to rapidly roll-out, scale up and maximize on coverage of the recently developed highly effective HPV vaccines in preventing HPV acquisition, crucial to decreasing morbidity and mortality [32].

## Supporting Information

S1 Dataset S1 Dataset in Microsoft Excel format. (XLSX)

S2 Dataset S2 Dataset in PDF format. (PDF)

S1 Fig Distribution of HPV genotypes in young women in KwaZulu-Natal, South Africa (high risk types 16, 18, 31, 33, 35, 39, 45, 51, 52, 56 and 58 highlighted in darker colour). (A) % of women infected. (B) HPV genotype.
(TIF)

S2 Fig Distribution of multiple concurrent HPV genotype infections in young women in KwaZulu-Natal, South Africa. (A) % of women infected. (B) Number of HPV types.
(TIF)
