# Supplementary material for: High Burden of Human Papillomavirus (HPV) Infection among Young Women in KwaZulu-Natal, South Africa
Source: PLoS One. 2016 Jan 19;11(1):e0146603. doi: 10.1371/journal.pone.0146603 (PMC4718633; doi:10.1371/journal.pone.0146603)
Supplement: S2 Dataset — (PDF) [file pone.0146603.s002.pdf]

Subject	HPV	HPV6	HPV11	HPV16	HPV18	HPV26	HPV31	HPV33	HPV35	HPV39	HPV40	HPV42	HPV45	HPV51	HPV52	HPV53	HPV54	HPV55	HPV56	HPV58	HPV59	HPV61	HPV62	HPV66
1	1	1	1	0	0	0	0	0	0	0	1	0	0	0	0	0	0	0	0	1	0	1	1	0
2	1	0	0	0	0	0	0	0	0	0	0	0	0	0	0	0	0	0	0	0	0	0	0	0
3	1	0	0	1	0	0	0	0	0	0	0	0	0	0	1	0	0	0	0	0	0	1	0	0
4	0	0	0	0	0	0	0	0	0	0	0	0	0	0	0	0	0	0	0	0	0	0	0	0
5	1	0	0	0	0	0	0	0	1	0	0	0	0	1	0	1	0	0	0	0	0	0	1	0
6	1	0	0	0	0	0	0	0	0	0	0	0	0	0	0	0	0	0	0	0	0	0	0	0
7	0	0	0	0	0	0	0	0	0	0	0	0	0	0	0	0	0	0	0	0	0	0	0	0
8	0	0	0	0	0	0	0	0	0	0	0	0	0	0	0	0	0	0	0	0	0	0	0	0
9	1	0	0	0	1	0	0	0	0	0	0	0	0	0	0	0	0	0	0	0	0	0	1	0
10	1	0	0	0	0	0	0	0	0	0	0	0	0	0	0	0	0	0	0	0	0	0	0	0
11	0	0	0	0	0	0	0	0	0	0	0	0	0	0	0	0	0	0	0	0	0	0	0	0
12	1	0	0	0	0	0	0	1	0	0	0	0	0	1	0	0	0	0	0	1	0	0	1	0
13	1	0	0	0	0	1	0	0	0	0	0	0	0	0	0	0	0	0	0	0	0	0	0	0
14	0	0	0	0	0	0	0	0	0	0	0	0	0	0	0	0	0	0	0	0	0	0	0	0
15	1	0	0	0	0	0	0	0	0	0	0	0	1	0	0	1	0	0	1	0	0	0	1	1
16	1	0	0	0	0	0	0	1	0	0	0	0	0	0	0	0	0	0	0	0	0	0	0	1
17	1	0	0	1	0	0	0	0	0	0	0	0	0	0	0	0	0	0	0	0	0	0	0	0
18	0	0	0	0	0	0	0	0	0	0	0	0	0	0	0	0	0	0	0	0	0	0	0	0
19	0	0	0	0	0	0	0	0	0	0	0	0	0	0	0	0	0	0	0	0	0	0	0	0
20	1	0	0	0	0	0	0	0	0	0	0	0	0	0	1	0	0	0	1	0	0	0	0	0
21	1	0	0	0	0	0	0	0	0	0	0	0	0	1	0	0	0	0	0	0	1	0	0	0
22	0	0	0	0	0	0	0	0	0	0	0	0	0	0	0	0	0	0	0	0	0	0	0	0
23	1	0	0	0	0	0	0	0	0	0	0	0	0	0	0	0	0	0	0	0	0	1	0	0
24	1	0	0	0	1	0	0	0	0	1	0	0	1	0	0	0	0	0	0	1	0	0	0	1
25	1	0	0	0	0	0	0	0	0	0	0	0	0	1	0	0	0	0	0	0	0	0	0	0
26	0	0	0	0	0	0	0	0	0	0	0	0	0	0	0	0	0	0	0	0	0	0	0	0
27	1	0	0	0	1	0	0	0	0	0	0	0	0	0	0	0	0	0	0	0	0	0	0	0
28	0	0	0	0	0	0	0	0	0	0	0	0	0	0	0	0	0	0	0	0	0	0	0	0
29	1	0	0	1	1	0	0	0	0	0	0	0	0	0	0	0	0	1	0	0	0	0	0	0
30	0	0	0	0	0	0	0	0	0	0	0	0	0	0	0	0	0	0	0	0	0	0	0	0
31	0	0	0	0	0	0	0	0	0	0	0	0	0	0	0	0	0	0	0	0	0	0	0	0
32	1	0	0	0	0	0	0	0	0	1	0	0	0	0	0	1	0	0	0	0	1	0	0	1
33	0	0	0	0	0	0	0	0	0	0	0	0	0	0	0	0	0	0	0	0	0	0	0	0
34	0	0	0	0	0	0	0	0	0	0	0	0	0	0	0	0	0	0	0	0	0	0	0	0
35	1	0	0	1	0	0	0	0	0	0	0	0	0	0	0	0	0	0	0	0	0	0	0	0
36	0	0	0	0	0	0	0	0	0	0	0	0	0	0	0	0	0	0	0	0	0	0	0	0
37	1	0	0	0	0	0	0	0	1	0	0	0	0	1	0	0	0	0	0	0	0	0	0	0
38	1	0	0	0	0	0	0	0	0	0	0	0	0	0	0	0	0	0	0	0	0	0	0	0
39	1	1	0	0	0	0	0	0	0	0	0	0	0	0	0	0	0	0	0	0	0	0	0	0
40	1	0	0	0	0	0	0	0	0	1	0	0	0	0	0	0	0	0	0	0	0	0	0	1
41	1	1	0	0	0	0	0	0	1	0	0	0	0	0	0	0	0	0	0	0	0	0	0	0
42	0	0	0	0	0	0	0	0	0	0	0	0	0	0	0	0	0	0	0	0	0	0	0	0

Subject	HPV68	HPV69	HPV70	HPV71	HPV72	HPV73	HPV81	HPV82	HPV83	HPV84	HPV89	Age (years)	Age group (years)	Partner has other partners	Living with partner	Site	Injectable contraceptive	Injectable contraceptive type	Condom use
1	0	0	0	0	1	1	1	0	1	0	0	18	18+	Don't know	No	Rural	Yes	DMPA	Yes
2	0	0	0	1	0	0	0	0	0	0	0	22	18+	Yes	No	Rural	No	None	No
3	0	0	1	0	0	0	0	0	0	0	0	17	<18	No	No	Rural	No	None	No
4	0	0	0	0	0	0	0	0	0	0	0	19	18+	Don't know	No	Rural	Yes	DMPA	No
5	0	0	0	0	0	0	0	0	0	0	0	16	<18	Don't know	No	Rural	No	None	No
6	0	0	0	0	0	1	0	0	0	0	0	27	18+	No	No	Rural	Yes	DMPA	Yes
7	0	0	0	0	0	0	0	0	0	0	0	16	<18	No	No	Rural	Yes	DMPA	Yes
8	0	0	0	0	0	0	0	0	0	0	0	19	18+	No	No	Rural	Yes	Nuristerate	No
9	0	0	0	0	0	0	0	0	0	0	0	18	18+	Don't know	No	Rural	No	None	Yes
10	0	0	0	0	1	0	0	0	0	1	0	23	18+	No	No	Rural			
11	0	0	0	0	0	0	0	0	0	0	0	24	18+	No	No	Rural	Yes	Nuristerate	No
12	1	0	0	0	0	0	0	0	0	0	0	24	18+	Don't know	No	Rural	Yes	DMPA	Yes
13	0	0	0	0	0	0	0	0	0	0	0	19	18+	Don't know	No	Rural	No	None	No
14	0	0	0	0	0	0	0	0	0	0	0	21	18+	Don't know	No	Rural	Yes	DMPA	Yes
15	0	0	0	0	0	0	1	0	0	0	0	23	18+	No	No	Rural	Yes	DMPA	No
16	0	0	0	0	0	0	0	0	0	0	0	21	18+	Don't know	No	Rural	Yes	DMPA	Yes
17	0	0	0	0	0	0	0	0	0	0	0	21	18+	Yes	Yes	Rural	Yes	DMPA	No
18	0	0	0	0	0	0	0	0	0	0	0	17	<18	Don't know	No	Rural	Yes	DMPA	Yes
19	0	0	0	0	0	0	0	0	0	0	0	23	18+	Don't know	No	Rural	Yes	DMPA	No
20	0	0	0	0	0	0	0	0	0	0	0	19	18+	Don't know	No	Rural	Yes	DMPA	Yes
21	0	0	1	0	0	0	0	0	0	0	0	26	18+	No	No	Rural	No	None	Yes
22	0	0	0	0	0	0	0	0	0	0	0	29	18+	No	No	Rural	Yes	DMPA	No
23	0	0	0	0	0	0	0	0	0	0	0	15	<18	Don't know	No	Rural	Yes		Yes
24	1	0	0	0	0	0	0	0	0	0	0	24	18+	Don't know	No	Rural	Yes	DMPA	Yes
25	0	0	0	0	0	0	0	0	0	0	0	19	18+	No	No	Rural	Yes	DMPA	Yes
26	0	0	0	0	0	0	0	0	0	0	0	16	<18	No	No	Rural	No	None	Yes
27	0	0	0	0	0	0	0	1	1	0	0	19	18+	Don't know	No	Rural	No	None	Yes
28	0	0	0	0	0	0	0	0	0	0	0	21	18+	No	No	Rural	Yes	DMPA	Yes
29	0	0	1	0	0	0	0	0	0	0	0	18	18+	Don't know	No	Rural	Yes	DMPA	No
30	0	0	0	0	0	0	0	0	0	0	0	22	18+	Don't know	Yes	Rural	No	None	No
31	0	0	0	0	0	0	0	0	0	0	0	22	18+	Yes	No	Rural	No	None	No
32	0	0	0	0	0	0	0	0	0	0	0	25	18+	Don't know	No	Rural	Yes	DMPA	No
33	0	0	0	0	0	0	0	0	0	0	0	18	18+	Don't know	No	Rural	Yes	DMPA	No
34	0	0	0	0	0	0	0	0	0	0	0	18	18+	Don't know	No	Rural	Yes	DMPA	No
35	0	0	0	0	0	0	0	0	0	0	0	19	18+	Yes	Yes	Rural	No	None	Yes
36	0	0	0	0	0	0	0	0	0	0	0	16	<18	Don't know	No	Rural	No	None	Yes
37	1	0	0	0	0	0	0	0	0	0	0	21	18+	No	No	Rural	Yes	DMPA	No
38	0	0	0	0	0	0	0	0	1	0	0	27	18+	Don't know	No	Rural	No	None	No
39	0	0	0	0	0	0	0	0	1	0	0	28	18+	Yes	No	Rural	Yes	DMPA	Yes
40	0	0	0	0	0	0	0	0	0	0	0	27	18+	Don't know	Yes	Rural	No	None	Yes
41	0	0	1	0	0	0	0	0	0	0	0	18	18+	Don't know	No	Rural	Yes	DMPA	Yes
42	0	0	0	0	0	0	0	0	0	0	0	22	18+	Don't know	No	Rural			

Subject	Vaginal insertion practices	Abnormal pelvic exam	Relationship status	Number of sexual partners in the last 3 months
1	No	No	Stable/Married	1 partner
2	No	Yes	Stable/Married	1 partner
3	No	No	Stable/Married	1 partner
4	No	No	Stable/Married	1 partner
5	No	No	Stable/Married	1 partner
6	No	No	Stable/Married	1 partner
7	No	No	Stable/Married	1 partner
8	No	No	Stable/Married	1 partner
9	No	Yes	Stable/Married	1 partner
10	No	Yes	Stable/Married	1 partner
11	No	Yes	Stable/Married	1 partner
12	No	No	Stable/Married	1 partner
13	Yes	No	Stable/Married	1 partner
14	No	No	Stable/Married	1 partner
15	Yes	No	Stable/Married	1 partner
16	Yes	Yes	Stable/Married	1 partner
17	No	No	Stable/Married	1 partner
18	No	No	Stable/Married	1 partner
19	No	No	Stable/Married	1 partner
20	No	No	Stable/Married	1 partner
21	Yes	No	Stable/Married	1 partner
22	No	No	Single	1 partner
23	No	No	Stable/Married	1 partner
24	No	No	Stable/Married	1 partner
25	No	No	Stable/Married	1 partner
26	No	No	Single	1 partner
27	No	No	Stable/Married	1 partner
28	No	No	Single	1 partner
29	No	Yes	Stable/Married	1 partner
30	Yes	No	Single	1 partner
31	Yes	Yes	Stable/Married	1 partner
32	Yes	No	Stable/Married	1 partner
33	No	No	Stable/Married	1 partner
34	No	No	Stable/Married	1 partner
35	No	No	Single	1 partner
36	Yes	No		1 partner
37	Yes	No	Single	1 partner
38	Yes	No	Stable/Married	1 partner
39	No	Yes	Single	1 partner
40	No	No	Stable/Married	1 partner
41	No	No	Stable/Married	1 partner
42	No	No	Stable/Married	1 partner

Subject	HPV	HPV6	HPV11	HPV16	HPV18	HPV26	HPV31	HPV33	HPV35	HPV39	HPV40	HPV42	HPV45	HPV51	HPV52	HPV53	HPV54	HPV55	HPV56	HPV58	HPV59	HPV61	HPV62	HPV66
43	1	0	0	0	0	0	0	0	0	1	0	0	0	0	0	0	0	1	0	0	0	0	0	0
44	0	0	0	0	0	0	0	0	0	0	0	0	0	0	0	0	0	0	0	0	0	0	0	0
45	1	0	0	0	1	0	0	0	0	0	0	0	1	0	0	0	0	0	0	0	0	1	1	0
46	0	0	0	0	0	0	0	0	0	0	0	0	0	0	0	0	0	0	0	0	0	0	0	0
47	0	0	0	0	0	0	0	0	0	0	0	0	0	0	0	0	0	0	0	0	0	0	0	0
48	1	0	1	0	0	0	0	0	0	0	0	1	0	0	0	0	1	0	0	1	0	1	0	0
49	1	0	0	1	0	0	0	0	0	0	0	0	0	0	1	0	0	0	0	0	0	0	0	0
50	1	0	0	0	0	0	0	0	0	1	0	0	0	0	1	0	0	0	0	0	0	0	0	0
51	1	1	0	0	0	0	0	0	0	0	0	0	0	0	0	0	0	0	0	0	0	0	1	0
52	1	0	0	0	0	0	0	0	0	0	0	0	0	0	0	0	0	0	0	1	0	0	0	0
53	1	0	0	0	0	0	0	0	0	0	0	0	0	0	0	0	0	0	0	1	0	0	0	0
54	1	0	0	0	0	0	0	0	0	0	0	0	0	0	0	0	0	0	0	0	0	0	0	0
55	1	0	0	0	0	0	1	0	0	0	0	0	0	0	0	0	0	0	0	1	0	1	0	0
56	1	0	0	0	0	0	0	0	0	0	0	1	0	0	0	0	0	0	0	1	0	0	0	0
57	1	0	0	0	0	0	0	0	0	0	0	0	0	0	0	0	0	0	0	1	0	0	0	0
58	1	0	0	1	0	0	0	0	0	0	0	0	0	0	0	0	0	0	0	0	1	0	1	0
59	1	0	0	0	0	0	0	0	0	0	0	0	0	0	0	0	0	0	0	0	0	0	1	0
60	1	0	0	0	0	0	0	0	0	0	0	1	0	0	0	1	0	1	0	1	0	0	1	0
61	0	0	0	0	0	0	0	0	0	0	0	0	0	0	0	0	0	0	0	0	0	0	0	0
62	1	0	0	0	0	0	0	0	0	0	0	0	0	0	0	0	0	0	0	1	0	0	0	0
63	1	0	0	0	0	0	0	0	0	0	0	0	0	0	1	0	0	0	0	0	0	0	0	0
64	1	1	0	0	0	0	0	0	0	0	0	0	1	0	0	1	1	0	0	0	0	0	0	1
65	1	1	0	0	0	0	0	0	0	0	0	0	0	0	0	0	0	0	0	0	0	1	0	0
66	1	0	0	0	0	0	0	0	0	0	0	0	1	0	0	0	0	0	0	0	0	0	0	0
67	1	0	0	0	0	0	0	0	0	0	0	0	0	0	0	1	0	0	0	1	0	0	0	0
68	1	0	1	1	1	0	1	0	0	0	0	0	0	0	1	0	1	0	1	1	0	0	1	0
69	1	0	0	0	0	0	0	0	0	0	0	0	0	1	0	0	0	0	0	1	0	0	0	0
70	1	0	0	0	0	0	0	0	0	0	0	0	0	0	1	0	0	0	0	0	0	0	0	0
71	1	0	0	0	1	0	0	0	0	0	0	0	0	0	1	1	0	0	0	0	0	0	0	0
72	1	0	0	0	0	0	0	0	0	0	0	0	0	1	0	0	0	0	0	0	0	0	0	0
73	1	0	0	0	0	0	0	0	0	0	0	0	0	0	0	1	0	1	0	0	0	0	0	0
74	1	1	0	0	0	1	0	0	0	0	0	0	0	1	0	0	0	0	0	0	0	0	0	0
75	0	0	0	0	0	0	0	0	0	0	0	0	0	0	0	0	0	0	0	0	0	0	0	0
76	1	0	0	1	1	0	0	1	0	1	0	1	0	0	0	0	0	0	1	0	0	0	0	0
77	1	0	0	0	0	0	0	0	0	0	0	0	0	0	0	1	0	0	0	0	0	1	1	0
78	1	0	0	1	0	0	0	0	0	1	0	0	0	0	0	0	0	1	0	0	0	0	0	0
79	1	0	0	0	1	0	0	0	0	0	0	0	0	1	0	0	0	0	0	1	1	0	1	0
80	1	0	0	0	0	1	0	0	1	0	1	1	0	1	0	0	0	0	0	0	0	0	0	0
81	1	0	0	0	0	0	0	0	0	0	0	0	0	0	0	0	0	0	0	1	0	0	0	0
82	0	0	0	0	0	0	0	0	0	0	0	0	0	0	0	0	0	0	0	0	0	0	0	0
83	1	0	0	1	0	0	0	0	0	0	0	0	0	1	1	0	0	0	0	0	0	0	0	0
84	0	0	0	0	0	0	0	0	0	0	0	0	0	0	0	0	0	0	0	0	0	0	0	0

Subject	HPV68	HPV69	HPV70	HPV71	HPV72	HPV73	HPV81	HPV82	HPV83	HPV84	HPV89	Age (years)	Age group (years)	Partner has other partners	Living with partner	Site	Injectable contraceptive	Injectable contraceptive type	Condom use
43	0	0	0	0	1	1	1	0	0	0	0	18	18+	Don't know	No	Rural	Yes	DMPA	No
44	0	0	0	0	0	0	0	0	0	0	0	16	<18	Don't know	No	Rural	No	None	No
45	0	0	0	0	0	0	0	0	0	0	0	24	18+	Don't know	No	Rural	Yes	DMPA	No
46	0	0	0	0	0	0	0	0	0	0	0	16	<18	Don't know	No	Rural	Yes	DMPA	No
47	0	0	0	0	0	0	0	0	0	0	0	21	18+	Yes	No	Rural	Yes	DMPA	No
48	0	0	0	0	0	0	0	0	0	0	0	18	18+	Don't know	No	Rural	Yes	DMPA	No
49	0	0	0	0	0	0	0	0	0	0	0	19	18+	No	No	Rural	No	None	No
50	0	0	0	0	0	0	0	0	0	0	0	18	18+	Don't know	No	Rural	No	None	Yes
51	0	0	0	0	0	0	0	0	0	0	0	17	<18	Don't know	No	Rural	No	None	No
52	0	0	0	0	0	0	0	0	0	0	0	15	<18	Don't know	No	Rural	No	None	No
53	0	1	0	0	0	0	0	0	0	1	0	24	18+	No	No	Rural	Yes	DMPA	No
54	0	1	0	0	0	0	0	0	0	0	0	20	18+	Don't know	No	Rural	No	None	No
55	0	0	0	0	0	0	0	0	0	0	0	20	18+	Don't know	No	Rural	Yes	DMPA	Yes
56	0	0	1	0	0	0	0	0	0	0	0	18	18+	Don't know	No	Rural	No	None	No
57	0	0	0	0	0	0	0	0	0	0	0	19	18+	Don't know	No	Rural			
58	0	0	0	0	0	0	0	0	0	0	0	22	18+	Don't know	No	Rural	No	None	Yes
59	0	0	0	0	0	0	0	0	1	0	0	17	<18	Don't know	No	Rural	No	None	No
60	0	0	0	0	1	0	0	0	0	0	0	18	18+	Don't know	No	Rural	No	None	Yes
61	0	0	0	0	0	0	0	0	0	0	0	28	18+	No	Yes	Urban	No	None	No
62	0	0	0	0	0	0	0	0	0	0	1	23	18+	Don't know	No	Urban	Yes	Nuristerate	Yes
63	0	0	0	1	0	0	0	0	0	0	0	21	18+	Don't know	No	Urban	No	None	Yes
64	0	0	0	0	0	0	0	0	0	0	0	24	18+	Don't know	No	Urban	No	None	No
65	0	0	1	0	0	0	0	0	0	0	0	25	18+	Yes	Yes	Urban	Yes	DMPA	Yes
66	0	0	0	0	0	0	0	0	0	0	0	16	<18	Don't know	No	Urban	No	None	Yes
67	0	0	0	0	1	0	0	0	0	0	0	21	18+	No	No	Urban	No	None	Yes
68	0	1	0	0	0	0	0	0	0	0	1	19	18+	Don't know	No	Urban	No	None	Yes
69	0	0	0	0	0	0	0	0	0	0	0	16	<18	Don't know	No	Urban	No	None	No
70	0	0	0	0	0	0	0	0	0	0	0	14	<18	Don't know	No	Urban	No	None	Yes
71	0	0	0	0	0	0	1	0	1	0	0	19	18+	Don't know	No	Urban	No	None	Yes
72	1	0	0	0	0	0	0	0	0	0	0	27	18+	Yes	No	Urban	Yes	DMPA	No
73	0	0	0	0	0	0	0	0	0	0	0	25	18+	Don't know	No	Urban	No	None	Yes
74	0	0	0	0	0	0	0	0	0	1	0	22	18+	Don't know	No	Urban	No	None	Yes
75	0	0	0	0	0	0	0	0	0	0	0	26	18+	Don't know	No	Urban	No	None	Yes
76	0	0	0	0	0	1	0	0	0	0	0	18	18+	No	No	Urban	No	None	Yes
77	0	0	0	1	0	0	0	0	0	0	0	27	18+	Don't know	No	Urban	No	None	Yes
78	0	0	0	0	1	0	1	0	1	0	0	23	18+	No	No	Urban	No	None	No
79	0	1	0	0	0	0	0	0	0	0	0	20	18+	No	No	Urban	No	None	Yes
80	1	0	0	1	0	0	0	1	0	1	0	20	18+	Yes	No	Urban	No	None	No
81	0	0	0	0	0	0	1	0	1	0	0	21	18+	No	No	Urban	No	None	Yes
82	0	0	0	0	0	0	0	0	0	0	0	20	18+	No	Yes	Urban	No	None	Yes
83	0	0	0	0	0	0	0	0	0	0	0	21	18+	Don't know	No	Urban	No	None	No
84	0	0	0	0	0	0	0	0	0	0	0	22	18+	Don't know	No	Urban	No	None	Yes

Subject	Vaginal insertion practices	Abnormal pelvic exam	Relationship status	Number of sexual partners in the last 3 months
43	No	No	Single	1 partner
44	No	Yes	Stable/Married	1 partner
45	No	No	Stable/Married	1 partner
46	No	Yes	Stable/Married	1 partner
47	No	No	Single	1 partner
48	No	Yes	Stable/Married	1 partner
49	No	Yes	Single	1 partner
50	Yes	No	Stable/Married	1 partner
51	No	No	Stable/Married	1 partner
52	Yes	No	Stable/Married	1 partner
53	No	Yes	Stable/Married	1 partner
54	No	Yes	Stable/Married	1 partner
55	No	Yes	Stable/Married	1 partner
56	No	Yes	Stable/Married	1 partner
57	Yes	No	Stable/Married	
58	Yes	No	Stable/Married	1 partner
59	Yes	No	Stable/Married	2+ partners
60	No	Yes	Stable/Married	1 partner
61	Yes	No	Stable/Married	1 partner
62	Yes	Yes	Stable/Married	1 partner
63	Yes	Yes	Stable/Married	1 partner
64	No	Yes	Stable/Married	1 partner
65	Yes	Yes	Stable/Married	1 partner
66	No	Yes	Stable/Married	1 partner
67	Yes	No	Stable/Married	1 partner
68	Yes	Yes	Stable/Married	1 partner
69	Yes	Yes	Stable/Married	1 partner
70	Yes	Yes	Stable/Married	2+ partners
71	Yes	Yes	Stable/Married	1 partner
72	Yes	No	Stable/Married	1 partner
73	Yes	No	Stable/Married	1 partner
74	Yes	Yes	Stable/Married	1 partner
75	Yes	No	Single	1 partner
76	Yes	No	Single	2+ partners
77	Yes	No	Stable/Married	1 partner
78	Yes	No	Stable/Married	1 partner
79	No	No	Stable/Married	1 partner
80	Yes	Yes	Stable/Married	1 partner
81	Yes	Yes	Stable/Married	2+ partners
82	No	No	Stable/Married	1 partner
83	No	Yes	Stable/Married	1 partner
84	No	Yes	Stable/Married	1 partner

Subject	HPV	HPV6	HPV11	HPV16	HPV18	HPV26	HPV31	HPV33	HPV35	HPV39	HPV40	HPV42	HPV45	HPV51	HPV52	HPV53	HPV54	HPV55	HPV56	HPV58	HPV59	HPV61	HPV62	HPV66
85	1	0	1	0	0	0	0	0	0	0	0	0	0	0	0	0	0	0	0	0	0	0	0	0
86	1	0	0	0	0	0	0	0	0	0	0	0	0	0	1	0	0	0	0	0	0	0	0	0
87	1	0	0	0	0	0	0	0	0	0	0	0	0	1	0	0	0	0	0	0	0	0	1	0
88	1	0	0	0	0	0	0	1	0	0	0	0	0	0	0	0	0	0	0	0	0	0	0	0
89	1	0	0	0	0	0	0	0	0	0	0	0	0	0	0	0	0	0	0	0	0	0	0	0
90	1	0	0	0	1	0	0	0	1	1	0	0	1	0	0	1	0	0	0	0	1	1	0	0
91	1	0	0	0	0	0	1	0	0	0	0	0	0	0	0	0	0	0	0	0	0	0	0	0
92	1	0	0	0	0	0	0	0	0	0	0	0	0	0	0	0	0	1	0	0	0	0	0	0
93	1	0	0	0	0	0	0	0	0	0	0	0	0	0	0	0	0	0	0	0	0	0	1	0
94	1	0	0	0	0	0	0	0	0	0	0	0	0	0	0	0	0	0	0	0	0	0	1	0
95	0	0	0	0	0	0	0	0	0	0	0	0	0	0	0	0	0	0	0	0	0	0	0	0
96	1	0	0	0	0	0	0	1	0	0	0	1	0	0	0	0	0	0	0	0	0	0	0	0
97	1	0	1	1	0	1	0	0	1	0	0	0	0	0	1	0	0	0	0	1	0	0	0	0
98	1	0	0	0	0	0	0	0	0	0	0	0	0	0	0	0	0	0	0	0	1	1	0	0
99	1	0	0	0	0	0	0	0	0	0	0	0	0	0	0	0	0	0	0	0	1	0	0	0
100	1	0	0	0	0	0	0	0	0	0	0	0	0	1	0	0	0	0	0	0	0	0	0	0
101	1	1	0	0	0	0	0	0	0	0	0	0	0	1	0	0	0	0	0	0	0	1	0	0
102	1	0	0	0	0	0	0	0	0	0	0	0	0	0	0	0	0	0	0	0	0	0	0	0
103	1	0	0	0	0	0	0	0	0	0	0	0	0	1	0	0	0	0	0	0	0	0	0	0
104	1	1	0	1	0	0	0	0	0	0	0	0	0	0	0	0	0	0	0	0	0	0	0	0
105	1	0	0	0	0	0	0	0	0	0	0	0	0	0	0	0	0	0	0	0	0	0	0	0
106	1	0	0	0	1	0	1	0	0	1	0	0	1	1	0	1	1	0	1	1	0	0	1	1
107	1	0	0	0	0	0	0	0	0	0	0	0	0	0	0	0	0	0	0	0	0	0	0	0
108	0	0	0	0	0	0	0	0	0	0	0	0	0	0	0	0	0	0	0	0	0	0	0	0
109	0	0	0	0	0	0	0	0	0	0	0	0	0	0	0	0	0	0	0	0	0	0	0	0
110	1	0	0	0	0	0	0	0	0	1	0	0	0	0	1	0	0	1	0	1	1	0	0	0
111	1	0	0	0	0	0	0	0	0	0	0	0	0	0	0	0	0	0	0	1	0	0	0	0
112	1	0	0	0	0	0	0	0	1	1	0	0	1	1	0	0	1	0	1	0	0	0	1	0
113	1	0	0	0	0	0	0	0	0	0	0	0	0	1	0	0	0	0	0	0	0	0	0	0
114	1	0	0	1	0	0	0	0	0	0	0	0	0	0	0	0	1	0	0	1	1	0	0	1
115	0	0	0	0	0	0	0	0	0	0	0	0	0	0	0	0	0	0	0	0	0	0	0	0
116	1	1	0	0	0	0	0	0	0	0	0	0	1	0	0	0	0	0	1	0	1	0	0	1
117	0	0	0	0	0	0	0	0	0	0	0	0	0	0	0	0	0	0	0	0	0	0	0	0
118	1	0	0	1	0	1	0	0	0	0	0	0	0	0	0	0	0	0	0	0	0	0	1	0
119	1	0	0	0	0	0	0	0	1	0	0	0	0	0	0	0	0	0	0	0	0	0	0	0
120	1	0	0	0	0	0	0	0	0	0	0	0	0	0	0	0	0	0	0	0	1	0	0	0
121	0	0	0	0	0	0	0	0	0	0	0	0	0	0	0	0	0	0	0	0	0	0	0	0
122	1	0	0	0	0	0	0	0	1	0	0	0	0	0	0	1	0	0	0	0	0	0	0	0
123	1	0	0	0	1	0	0	0	0	0	0	0	0	0	0	0	0	1	0	0	0	0	0	0
124	1	0	0	0	0	0	0	0	0	0	0	0	0	0	1	0	0	0	0	0	0	0	0	0
125	1	0	0	0	1	0	0	0	0	0	0	0	0	0	0	0	0	0	0	0	1	0	0	0
126	0	0	0	0	0	0	0	0	0	0	0	0	0	0	0	0	0	0	0	0	0	0	0	0

Subject	HPV68	HPV69	HPV70	HPV71	HPV72	HPV73	HPV81	HPV82	HPV83	HPV84	HPV89	Age (years)	Age group (years)	Partner has other partners	Living with partner	Site	Injectable contraceptive	Injectable contraceptive type	Condom use
85	0	0	0	0	0	0	0	0	0	0	0	20	18+	Don't know	No	Urban	Yes	Nuristerate	No
86	0	0	0	0	0	0	0	0	0	0	1	24	18+	No	No	Urban	No	None	Yes
87	0	1	0	0	0	0	0	0	0	0	0	25	18+	Yes	No	Urban	Yes	DMPA	No
88	0	0	1	0	0	0	0	0	0	0	0	23	18+	Don't know	No	Urban	No	None	No
89	1	0	0	0	0	0	0	0	0	0	0	26	18+	Yes	No	Urban	No	None	Yes
90	1	0	0	0	1	1	0	0	0	1	1	20	18+	Don't know	No	Urban	No	None	No
91	0	0	0	0	0	0	0	0	0	0	0	23	18+	Don't know	No	Urban	No	None	Yes
92	0	0	0	0	0	0	0	0	0	0	0	26	18+	No	Yes	Urban	No	None	Yes
93	0	0	0	0	0	0	0	0	0	0	0	28	18+	Don't know	No	Urban	Yes	Nuristerate	No
94	0	0	0	0	0	0	0	1	0	0	0	22	18+	Don't know	No	Urban	No	None	Yes
95	0	0	0	0	0	0	0	0	0	0	0	24	18+	Yes	No	Urban	No	None	No
96	0	0	0	0	0	0	0	0	0	0	0	20	18+	Don't know	No	Urban	Yes	Nuristerate	Yes
97	0	0	0	0	0	0	0	0	0	0	0	27	18+	Don't know	No	Urban	No	None	No
98	0	0	0	0	0	0	0	0	0	0	0	19	18+	Don't know	No	Urban	Yes	Nuristerate	No
99	0	0	1	0	0	0	0	0	0	0	0	27	18+	Yes	No	Urban	No	None	Yes
100	0	0	0	0	0	0	0	0	0	0	0	24	18+	Don't know	Yes	Urban	No	None	Yes
101	0	0	0	0	0	1	0	0	0	0	0	22	18+	No	No	Urban	No	None	No
102	0	0	0	0	0	1	0	0	1	0	1	24	18+	Don't know	No	Urban	Yes	DMPA	Yes
103	0	0	0	0	0	0	0	0	0	0	0	23	18+	Don't know	No	Urban	No	None	Yes
104	0	0	0	0	0	0	0	0	0	0	0	28	18+	Yes	No	Urban	No	None	Yes
105	0	0	0	0	0	0	1	0	0	0	0	23	18+	Don't know	No	Urban	No	None	Yes
106	0	0	0	0	0	1	0	0	0	0	0	22	18+	Don't know	No	Urban	No	None	No
107	0	0	0	1	0	0	0	0	0	0	0	28	18+	Don't know	Yes	Urban	No	None	Yes
108	0	0	0	0	0	0	0	0	0	0	0	23	18+	Don't know	Yes	Urban	Yes	DMPA	Yes
109	0	0	0	0	0	0	0	0	0	0	0	23	18+	No	Yes	Urban	No	None	Yes
110	0	0	0	0	0	0	0	0	0	0	0	24	18+	No	No	Urban	No	None	Yes
111	0	0	0	0	0	0	0	0	0	0	0	24	18+	Yes	No	Urban	Yes	Nuristerate	Yes
112	0	0	0	0	1	0	0	0	0	0	0	21	18+	Don't know	No	Urban	No	None	No
113	0	0	0	0	0	0	0	0	0	0	0	20	18+	Don't know	No	Urban	No	None	No
114	0	0	0	0	0	0	0	0	0	1	0	24	18+	Don't know	No	Urban	No	None	Yes
115	0	0	0	0	0	0	0	0	0	0	0	23	18+	Don't know	No	Urban	Yes	DMPA	No
116	0	0	0	0	0	0	0	0	0	1	0	19	18+	Don't know	No	Urban	No	None	No
117	0	0	0	0	0	0	0	0	0	0	0	26	18+	Yes	Yes	Urban	No	None	No
118	0	0	0	0	0	0	0	0	0	0	0	28	18+	Don't know	Yes	Urban	No	None	No
119	0	0	1	0	0	0	0	0	0	0	0	23	18+	Yes	No	Urban	No	None	No
120	0	0	0	0	0	0	0	0	0	0	0	21	18+	Yes	No	Urban	No	None	No
121	0	0	0	0	0	0	0	0	0	0	0	22	18+	No	Yes	Urban	No	None	Yes
122	0	0	0	0	0	0	0	0	0	0	0	25	18+	Don't know	Yes	Urban	No	None	Yes
123	0	0	0	0	0	0	1	0	0	0	0	22	18+	Don't know	No	Urban	No	None	Yes
124	0	0	0	0	0	0	0	0	0	0	0	20	18+	Yes	No	Urban	No	None	Yes
125	1	0	0	0	1	0	0	0	0	0	0	21	18+	Yes	No	Urban	Yes	Nuristerate	No
126	0	0	0	0	0	0	0	0	0	0	0	21	18+	Yes	No	Urban	No	None	Yes

Subject	Vaginal insertion practices	Abnormal pelvic exam	Relationship status	Number of sexual partners in the last 3 months
85	No	No	Stable/Married	1 partner
86	Yes	No	Stable/Married	1 partner
87	Yes	No	Stable/Married	2+ partners
88	Yes	No	Stable/Married	1 partner
89	No	No	Stable/Married	1 partner
90	Yes	No	Stable/Married	2+ partners
91	No	No	Stable/Married	1 partner
92	No	No	Stable/Married	2+ partners
93	No	Yes	Stable/Married	1 partner
94	Yes	No	Stable/Married	1 partner
95	No	No	Stable/Married	1 partner
96	No	Yes	Stable/Married	1 partner
97	Yes	No	Stable/Married	1 partner
98	No	Yes	Stable/Married	1 partner
99	Yes	No	Stable/Married	1 partner
100	Yes	No	Stable/Married	1 partner
101	No	No	Stable/Married	1 partner
102	No	No	Stable/Married	1 partner
103	Yes	No	Stable/Married	2+ partners
104	Yes	No	Single	1 partner
105	No	No	Single	1 partner
106	No	No	Stable/Married	1 partner
107	Yes	Yes	Stable/Married	1 partner
108	No	No	Stable/Married	1 partner
109	Yes	No	Stable/Married	1 partner
110	No	Yes	Stable/Married	1 partner
111	No	No	Stable/Married	1 partner
112	No	No	Stable/Married	1 partner
113	No	No	Stable/Married	1 partner
114	No	No	Stable/Married	1 partner
115	Yes	No	Stable/Married	1 partner
116	No	Yes	Stable/Married	1 partner
117	No	No	Stable/Married	1 partner
118	No	No	Stable/Married	1 partner
119	Yes	No	Stable/Married	2+ partners
120	No	Yes	Stable/Married	1 partner
121	No	No	Stable/Married	1 partner
122	No	No	Stable/Married	1 partner
123	Yes	Yes	Stable/Married	1 partner
124	No	No	Stable/Married	1 partner
125	No	No	Single	1 partner
126	No	No	Stable/Married	2+ partners

Subject	HPV	HPV6	HPV11	HPV16	HPV18	HPV26	HPV31	HPV33	HPV35	HPV39	HPV40	HPV42	HPV45	HPV51	HPV52	HPV53	HPV54	HPV55	HPV56	HPV58	HPV59	HPV61	HPV62	HPV66
127	1	0	0	0	0	0	0	0	0	0	0	0	0	0	0	0	0	0	0	0	0	0	1	0
128	0	0	0	0	0	0	0	0	0	0	0	0	0	0	0	0	0	0	0	0	0	0	0	0
129	1	1	0	0	0	0	0	0	1	1	0	0	1	0	0	1	0	0	0	1	0	0	0	1
130	1	0	0	1	0	0	0	0	0	0	0	0	0	0	0	0	0	0	0	0	0	0	0	0
131	1	0	0	0	0	0	0	0	1	0	0	0	1	0	0	0	0	0	0	0	0	0	0	0
132	1	0	0	0	0	0	0	0	0	0	0	0	0	0	1	0	0	1	0	0	0	0	0	0
133	1	0	0	0	0	0	0	0	0	0	0	0	1	0	0	0	0	0	0	0	0	1	0	0
134	1	0	0	0	0	0	0	0	0	0	0	0	0	0	0	1	0	0	0	0	0	1	0	0
135	1	0	0	0	0	0	0	0	0	0	0	0	0	0	0	0	1	0	0	0	0	0	1	0
136	1	0	0	0	0	0	0	0	0	0	0	0	0	0	0	0	0	0	0	1	0	0	0	0
137	1	0	0	0	0	0	0	0	0	0	0	0	1	1	0	0	0	0	0	0	0	0	0	0
138	1	0	0	1	0	0	0	0	0	1	0	0	0	0	0	1	1	0	0	1	1	0	0	0
139	1	0	0	0	0	0	1	0	1	0	0	0	0	0	0	0	0	0	0	0	0	0	0	0
140	1	1	0	0	0	0	0	0	0	0	0	0	0	1	1	0	1	0	0	0	0	0	0	0
141	1	0	0	0	1	0	0	0	0	1	0	0	0	1	1	0	0	0	0	0	0	0	1	0
142	1	0	0	0	0	0	0	0	0	1	0	0	0	0	0	1	0	1	0	0	1	0	0	0
143	1	0	0	0	0	0	0	0	0	0	0	1	0	0	0	0	0	0	0	0	0	0	0	0
144	1	0	0	0	1	0	0	0	0	0	0	0	0	0	0	1	0	0	0	0	0	0	0	0
145	1	0	0	0	0	0	0	0	0	1	0	0	1	0	0	0	0	0	0	0	0	0	1	0
146	1	0	0	0	0	1	0	0	0	1	0	0	1	0	0	0	0	0	0	0	0	0	0	0
147	1	0	0	1	0	0	1	0	0	1	0	0	0	0	0	0	0	0	0	0	0	0	0	0
148	0	0	0	0	0	0	0	0	0	0	0	0	0	0	0	0	0	0	0	0	0	0	0	0
149	1	0	0	1	0	0	0	0	0	0	0	0	1	0	0	0	0	0	0	1	0	0	0	0
150	0	0	0	0	0	0	0	0	0	0	0	0	0	0	0	0	0	0	0	0	0	0	0	0
151	0	0	0	0	0	0	0	0	0	0	0	0	0	0	0	0	0	0	0	0	0	0	0	0
152	1	0	0	0	0	1	0	0	0	0	0	0	0	0	0	0	0	0	0	0	0	1	0	0
153	1	0	0	0	0	0	0	0	0	0	0	0	0	0	0	1	0	0	0	0	0	0	0	0
154	1	0	0	0	0	0	0	0	0	1	0	0	0	0	0	0	0	0	0	0	0	0	0	0
155	1	0	0	0	0	0	0	0	0	0	0	0	0	0	0	1	0	0	0	0	1	0	0	0
156	0	0	0	0	0	0	0	0	0	0	0	0	0	0	0	0	0	0	0	0	0	0	0	0
157	0	0	0	0	0	0	0	0	0	0	0	0	0	0	0	0	0	0	0	0	0	0	0	0
158	1	0	0	0	0	0	0	0	0	0	0	0	0	0	0	0	0	0	0	0	0	0	0	1
159	1	1	0	0	0	1	0	0	0	1	0	0	0	0	1	0	0	0	0	0	0	1	0	0
160	0	0	0	0	0	0	0	0	0	0	0	0	0	0	0	0	0	0	0	0	0	0	0	0
161	1	0	0	0	0	0	0	0	0	0	0	0	0	0	0	0	1	0	0	0	0	0	0	0
162	0	0	0	0	0	0	0	0	0	0	0	0	0	0	0	0	0	0	0	0	0	0	0	0
163	0	0	0	0	0	0	0	0	0	0	0	0	0	0	0	0	0	0	0	0	0	0	0	0
164	0	0	0	0	0	0	0	0	0	0	0	0	0	0	0	0	0	0	0	0	0	0	0	0
165	1	0	0	1	0	0	0	0	0	0	0	0	0	0	0	0	0	0	0	0	0	0	0	0
166	1	0	0	1	0	0	0	0	0	0	0	0	0	0	0	0	0	1	0	0	0	0	0	0
167	1	0	0	0	0	0	0	0	0	0	0	0	0	0	1	0	0	0	0	0	1	1	0	0
168	0	0	0	0	0	0	0	0	0	0	0	0	0	0	0	0	0	0	0	0	0	0	0	0

Subject	HPV68	HPV69	HPV70	HPV71	HPV72	HPV73	HPV81	HPV82	HPV83	HPV84	HPV89	Age (years)	Age group (years)	Partner has other partners	Living with partner	Site	Injectable contraceptive	Injectable contraceptive type	Condom use
127	0	0	0	0	1	0	0	0	0	0	0	23	18+	Don't know	No	Urban	No	None	No
128	0	0	0	0	0	0	0	0	0	0	0	22	18+	Yes	No	Urban	No	None	No
129	0	0	0	0	0	0	0	0	0	0	0	21	18+	Yes	No	Urban	No	None	No
130	0	0	0	0	0	0	0	0	0	0	0	24	18+	Yes	No	Urban	No	None	No
131	0	0	0	0	0	0	1	0	0	0	0	23	18+	Don't know	Yes	Urban	No	None	No
132	0	0	0	0	1	0	0	0	0	0	0	21	18+	Don't know	No	Urban	No	None	No
133	1	0	0	0	0	0	0	0	0	0	0	21	18+	Don't know	No	Urban	No	None	No
134	0	0	0	0	0	0	0	0	0	0	0	26	18+	Don't know	Yes	Urban	Yes	DMPA	Yes
135	0	0	0	0	1	0	0	0	0	0	0	21	18+	No	No	Urban	No	None	Yes
136	0	0	0	0	0	0	0	0	0	0	0	23	18+	Don't know	No	Urban	No	None	Yes
137	0	0	0	0	0	0	0	0	0	0	0	19	18+	Don't know	No	Urban	No	None	No
138	1	0	1	0	0	0	0	0	0	0	0	21	18+	Yes	No	Urban	No	None	Yes
139	0	0	0	0	0	0	0	0	0	0	0	23	18+	Yes	No	Urban	No	None	No
140	0	0	0	0	0	0	0	0	0	1	1	23	18+	Don't know	No	Urban	Yes	DMPA	No
141	0	0	0	0	0	0	0	0	0	1	0	21	18+	Don't know	No	Urban	No	None	Yes
142	0	0	0	0	0	0	0	0	0	0	0	21	18+	No	No	Urban	No	None	Yes
143	0	0	0	0	0	0	0	0	0	0	0	24	18+	Don't know	No	Urban	No	None	No
144	0	0	0	1	0	0	0	0	0	0	0	25	18+	No	Yes	Urban	No	None	Yes
145	0	0	0	0	0	1	0	0	0	0	0	23	18+	No	Yes	Urban	No	None	No
146	0	0	0	0	0	0	0	0	0	0	1	21	18+	No	No	Urban	No	None	Yes
147	0	0	0	0	0	0	0	0	0	0	0	18	18+	Yes	No	Urban	No	None	No
148	0	0	0	0	0	0	0	0	0	0	0	20	18+	Don't know	No	Urban	No	None	Yes
149	0	0	1	0	1	0	0	0	0	0	0	19	18+	Don't know	No	Urban	Yes	DMPA	No
150	0	0	0	0	0	0	0	0	0	0	0	30	18+	Don't know	Yes	Urban	No	None	No
151	0	0	0	0	0	0	0	0	0	0	0	30	18+	Don't know	No	Urban	No	None	Yes
152	0	0	0	0	0	0	0	0	0	0	1	24	18+	Yes	No	Urban	No	None	Yes
153	0	0	0	0	0	0	0	0	0	0	1	21	18+	Don't know	Yes	Urban	No	None	Yes
154	0	0	0	0	0	0	0	0	0	0	0	30	18+	Don't know	No	Urban	No	None	Yes
155	0	0	0	0	0	1	0	0	1	1	0	20	18+	Yes	No	Urban	Yes	DMPA	Yes
156	0	0	0	0	0	0	0	0	0	0	0	21	18+	No	No	Rural	Yes	DMPA	No
157	0	0	0	0	0	0	0	0	0	0	0	28	18+	Yes	No	Rural	No	None	No
158	0	0	0	0	0	0	0	0	0	0	0	18	18+	Yes	No	Rural	Yes	DMPA	No
159	0	0	0	0	0	0	1	0	0	0	0	17	<18	Yes	No	Rural	No	None	No
160	0	0	0	0	0	0	0	0	0	0	0	19	18+	No	No	Rural	No	None	No
161	0	0	0	0	0	1	0	0	0	0	0	15	<18	Don't know	No	Rural	No	None	Yes
162	0	0	0	0	0	0	0	0	0	0	0	18	18+	Yes	No	Rural	Yes	DMPA	No
163	0	0	0	0	0	0	0	0	0	0	0	24	18+	No	Yes	Rural	No	None	Yes
164	0	0	0	0	0	0	0	0	0	0	0	26	18+	Yes	No	Rural	Yes	DMPA	No
165	0	0	0	0	0	0	0	0	0	0	0	20	18+	Yes	No	Rural	Yes	DMPA	Yes
166	0	0	0	0	0	1	0	0	0	0	0	19	18+	No	No	Rural	Yes	DMPA	No
167	0	0	1	0	0	0	0	0	0	0	0	19	18+	Don't know	No	Rural	No	None	No
168	0	0	0	0	0	0	0	0	0	0	0	17	<18	Yes	No	Rural	No	None	Yes

Subject	Vaginal insertion practices	Abnormal pelvic exam	Relationship status	Number of sexual partners in the last 3 months
127	No	Yes	Stable/Married	1 partner
128	No	No	Stable/Married	1 partner
129	No	Yes	Stable/Married	1 partner
130	No	No	Stable/Married	1 partner
131	No	Yes	Stable/Married	1 partner
132	Yes	No	Stable/Married	1 partner
133	No	No	Stable/Married	1 partner
134	No	No	Stable/Married	1 partner
135	No	Yes	Stable/Married	2+ partners
136	Yes	No	Stable/Married	1 partner
137	No	Yes	Stable/Married	1 partner
138	Yes	Yes	Stable/Married	2+ partners
139	No	Yes	Stable/Married	1 partner
140	No	Yes	Stable/Married	1 partner
141	No	Yes	Stable/Married	1 partner
142	Yes	No	Stable/Married	1 partner
143	No	No	Stable/Married	1 partner
144	Yes	Yes	Stable/Married	1 partner
145	Yes	No	Stable/Married	1 partner
146	Yes	Yes	Stable/Married	1 partner
147	No	Yes	Single	1 partner
148	No	Yes	Stable/Married	1 partner
149	Yes	No	Single	2+ partners
150	No	Yes	Stable/Married	1 partner
151	No	No	Single	2+ partners
152	No	No	Single	2+ partners
153	No	Yes	Stable/Married	2+ partners
154	Yes	No	Stable/Married	2+ partners
155	No	No	Stable/Married	2+ partners
156	No	Yes	Stable/Married	1 partner
157	Yes	Yes	Stable/Married	1 partner
158	No	Yes	Stable/Married	1 partner
159	Yes	Yes	Stable/Married	1 partner
160	No	No	Stable/Married	1 partner
161	No	Yes	Stable/Married	1 partner
162	No	Yes	Stable/Married	1 partner
163	No	Yes	Stable/Married	1 partner
164	Yes	Yes	Stable/Married	1 partner
165	Yes	Yes	Stable/Married	1 partner
166	No	Yes	Stable/Married	1 partner
167	No	Yes	Stable/Married	1 partner
168	No	No	Stable/Married	1 partner

Subject	HPV	HPV6	HPV11	HPV16	HPV18	HPV26	HPV31	HPV33	HPV35	HPV39	HPV40	HPV42	HPV45	HPV51	HPV52	HPV53	HPV54	HPV55	HPV56	HPV58	HPV59	HPV61	HPV62	HPV66
169	1	0	0	0	1	0	0	0	0	0	0	0	0	1	0	0	0	0	0	0	0	0	0	0
170	1	0	0	0	1	0	0	0	0	0	0	1	0	0	0	0	0	0	0	0	0	0	0	0
171	0	0	0	0	0	0	0	0	0	0	0	0	0	0	0	0	0	0	0	0	0	0	0	0
172	1	0	0	0	0	0	0	0	0	1	0	0	0	0	0	0	0	0	0	0	0	0	0	0
173	0	0	0	0	0	0	0	0	0	0	0	0	0	0	0	0	0	0	0	0	0	0	0	0
174	1	0	0	0	0	0	0	0	0	0	0	0	0	0	0	0	0	0	0	0	0	1	0	0
175	1	0	0	0	0	0	0	0	0	1	0	0	0	0	1	0	0	0	0	0	0	0	0	0
176	1	0	0	0	0	0	0	0	0	0	0	0	0	0	0	0	0	0	0	0	1	0	0	0
177	1	0	0	1	1	0	0	0	0	0	0	0	0	0	0	0	0	0	0	1	0	0	0	0
178	1	0	0	1	0	0	0	0	0	0	0	0	0	0	0	0	0	1	0	0	0	0	0	0
179	1	0	0	0	0	0	0	0	0	0	0	0	0	0	0	0	0	0	0	0	0	1	1	0
180	0	0	0	0	0	0	0	0	0	0	0	0	0	0	0	0	0	0	0	0	0	0	0	0
181	1	0	0	0	0	0	0	0	0	0	0	0	0	0	0	0	0	0	0	0	0	0	0	0
182	1	0	0	0	0	0	0	0	0	0	0	0	0	0	0	0	1	0	0	0	0	0	0	0
183	1	0	0	0	0	0	0	0	0	0	0	0	0	0	0	0	0	0	0	0	0	0	0	0
184	0	0	0	0	0	0	0	0	0	0	0	0	0	0	0	0	0	0	0	0	0	0	0	0
185	1	0	0	0	0	0	0	0	0	0	0	1	0	0	1	0	0	0	1	0	0	0	0	0
186	1	0	0	0	0	0	0	0	0	0	0	0	0	0	0	0	0	0	0	1	0	0	1	0
187	1	0	0	0	0	0	0	0	0	1	0	0	0	0	0	0	1	0	0	0	0	0	0	0
188	1	0	0	0	0	0	0	1	0	0	0	0	0	0	1	0	0	0	0	0	0	1	0	0
189	1	0	0	0	0	0	0	1	0	0	0	0	0	0	0	0	0	0	0	0	0	0	0	0
190	1	0	0	0	0	0	0	0	0	0	0	1	0	0	0	0	0	1	0	1	0	0	0	0
191	1	0	0	0	0	0	1	0	1	0	0	0	0	0	1	0	0	0	0	0	0	0	1	0
192	1	0	0	0	0	0	0	0	0	0	0	0	0	0	0	0	0	0	0	0	0	0	1	0
193	1	0	0	0	0	0	0	0	0	0	0	0	0	0	0	0	0	0	0	1	0	0	0	0
194	0	0	0	0	0	0	0	0	0	0	0	0	0	0	0	0	0	0	0	0	0	0	0	0
195	1	0	0	0	0	0	0	0	0	0	0	0	0	0	0	0	0	0	0	0	0	0	0	0
196	0	0	0	0	0	0	0	0	0	0	0	0	0	0	0	0	0	0	0	0	0	0	0	0
197	1	1	0	1	0	0	0	0	1	0	0	0	0	1	0	0	0	0	0	0	0	0	0	0
198	0	0	0	0	0	0	0	0	0	0	0	0	0	0	0	0	0	0	0	0	0	0	0	0
199	1	0	0	0	0	0	0	0	0	0	0	0	0	0	0	0	1	0	0	0	0	0	0	0
200	1	0	0	0	1	0	1	0	1	0	0	0	1	0	0	1	1	0	1	0	1	0	0	0
201	1	1	0	0	1	0	1	0	0	0	0	0	0	0	0	0	1	0	0	0	0	1	0	0
202	0	0	0	0	0	0	0	0	0	0	0	0	0	0	0	0	0	0	0	0	0	0	0	0
203	1	0	0	0	0	0	0	0	0	0	1	0	0	0	0	0	0	0	0	0	0	0	0	0
204	1	0	0	1	0	0	0	0	1	0	0	0	0	0	1	0	0	1	1	0	1	0	0	0
205	1	0	0	1	0	0	0	0	0	0	0	0	0	0	0	0	0	0	0	0	0	0	0	0
206	1	0	0	0	0	0	0	0	0	0	0	0	0	0	1	0	0	0	0	0	0	0	0	0
207	1	0	0	0	0	0	0	0	0	0	0	0	0	0	0	0	0	0	0	0	0	1	0	0
208	1	0	0	0	0	0	0	0	0	0	0	0	0	1	0	1	0	0	0	0	1	1	1	1
209	1	0	0	0	0	0	0	0	0	0	0	0	0	0	0	0	1	0	0	0	0	1	0	0
210	1	0	1	0	0	0	0	0	1	1	0	0	0	1	0	0	0	0	0	0	0	0	0	0

Subject	HPV68	HPV69	HPV70	HPV71	HPV72	HPV73	HPV81	HPV82	HPV83	HPV84	HPV89	Age (years)	Age group (years)	Partner has other partners	Living with partner	Site	Injectable contraceptive	Injectable contraceptive type	Condom use
169	0	0	0	0	0	0	0	0	0	0	0	18	18+	No	No	Rural	No	None	Yes
170	0	0	0	0	0	0	0	0	0	0	0	16	<18	Yes	No	Rural	No	None	Yes
171	0	0	0	0	0	0	0	0	0	0	0	17	<18	Don't know	No	Rural	No	None	Yes
172	0	0	0	0	0	0	0	0	0	0	0	18	18+	Don't know	No	Rural	No	None	Yes
173	0	0	0	0	0	0	0	0	0	0	0	20	18+	Yes	No	Rural	No	None	No
174	0	0	0	0	0	0	0	0	0	1	0	20	18+	Don't know	No	Rural	No	None	No
175	1	0	0	0	0	0	0	0	0	0	0	17	<18	No	No	Rural	No	None	No
176	0	0	0	0	0	0	0	0	0	0	0	18	18+	Don't know	No	Rural	No	None	Yes
177	0	0	0	0	0	0	1	1	0	0	0	20	18+	Don't know	No	Rural	No	None	Yes
178	0	0	1	0	0	1	1	0	0	0	0	14	<18	Yes	No	Rural	Yes	Nuristerate	Yes
179	1	0	0	0	0	0	1	0	0	0	0	17	<18	Yes	No	Rural	No	None	No
180	0	0	0	0	0	0	0	0	0	0	0	26	18+	Yes	No	Rural	No	None	No
181	1	0	0	0	0	1	1	0	0	0	0	16	<18	Don't know	No	Rural	No	None	No
182	0	0	0	0	0	0	0	0	0	0	0	26	18+	Yes	No	Rural	Yes	DMPA	No
183	0	0	0	0	1	0	0	0	0	0	0	21	18+	No	No	Rural	No	None	No
184	0	0	0	0	0	0	0	0	0	0	0	16	<18	No	No	Rural	No	None	Yes
185	0	0	0	0	0	0	0	0	0	0	0	20	18+	Yes	No	Rural	No	None	Yes
186	0	0	0	1	0	0	0	0	0	0	0	16	<18	Don't know	No	Rural	Yes	DMPA	Yes
187	0	0	1	0	0	0	0	0	0	0	0	19	18+	Don't know	No	Rural	No	None	Yes
188	0	0	0	0	0	0	0	0	0	0	0	16	<18	No	No	Rural	No	None	Yes
189	0	0	0	0	0	0	0	0	0	0	0	18	18+	Yes	No	Rural	No	None	No
190	1	0	1	0	0	0	0	0	1	0	0	19	18+	Don't know	No	Rural	No	None	Yes
191	0	0	0	0	0	0	1	0	0	0	0	19	18+	Don't know	No	Rural	No	None	No
192	0	0	0	0	0	0	0	0	0	0	0	17	<18	Yes	No	Rural	No	None	No
193	0	0	0	0	0	0	0	0	0	0	0	17	<18	Yes	No	Rural	No	None	No
194	0	0	0	0	0	0	0	0	0	0	0	15	<18	Yes	No	Rural	No	None	No
195	0	0	0	0	0	0	1	0	0	0	0	20	18+	No	No	Rural	No	None	No
196	0	0	0	0	0	0	0	0	0	0	0	23	18+	Don't know	No	Rural	Yes	DMPA	No
197	0	0	0	0	0	1	0	0	0	0	0	18	18+	Don't know	No	Rural	Yes	DMPA	Yes
198	0	0	0	0	0	0	0	0	0	0	0	19	18+	Don't know	No	Rural	No	None	Yes
199	0	0	1	0	0	0	0	0	0	0	0	18	18+	No	No	Rural	No	None	No
200	0	0	0	0	0	0	0	0	1	0	0	14	<18	No	No	Rural	No	None	No
201	0	0	0	0	0	0	0	0	0	1	0	15	<18	No	No	Rural	No	None	Yes
202	0	0	0	0	0	0	0	0	0	0	0	16	<18	Yes	No	Rural	No	None	No
203	0	0	0	0	0	0	0	0	0	0	0	18	18+	Don't know	No	Rural	No	None	Yes
204	0	0	0	0	0	0	0	0	0	0	0	15	<18	Don't know	No	Rural	No	None	No
205	0	0	0	0	0	0	0	0	0	0	0	25	18+	No	Yes	Rural	Yes	DMPA	No
206	0	0	0	0	0	0	0	0	0	0	0	23	18+	Yes	No	Rural	Yes	DMPA	No
207	0	0	0	0	0	0	0	0	0	0	0	26	18+	Yes	No	Rural	Yes	DMPA	No
208	0	0	0	0	0	0	0	0	0	0	0	17	<18	Yes	No	Rural	No	None	No
209	0	0	0	0	0	0	0	0	1	0	0	16	<18	Don't know	No	Rural	No	None	No
210	0	0	0	0	0	0	0	0	0	0	0	17	<18	Don't know	No	Rural	No	None	Yes

Subject	Vaginal insertion practices	Abnormal pelvic exam	Relationship status	Number of sexual partners in the last 3 months
169	No	No	Stable/Married	1 partner
170	No	Yes	Stable/Married	1 partner
171	No	Yes	Stable/Married	1 partner
172	No	Yes	Stable/Married	1 partner
173	No	Yes	Stable/Married	1 partner
174	No	Yes	Stable/Married	2+ partners
175	No	No	Stable/Married	1 partner
176	No	No	Stable/Married	1 partner
177	No	No	Stable/Married	1 partner
178	No	Yes	Stable/Married	1 partner
179	No	No	Stable/Married	1 partner
180	No	No	Stable/Married	1 partner
181	No	Yes	Stable/Married	1 partner
182	Yes	Yes	Stable/Married	1 partner
183	No	Yes	Stable/Married	1 partner
184	Yes	Yes	Stable/Married	1 partner
185	Yes	Yes	Stable/Married	1 partner
186	No	No	Stable/Married	1 partner
187	Yes	Yes	Stable/Married	1 partner
188	No	No	Stable/Married	1 partner
189	No	Yes	Stable/Married	1 partner
190	No	No	Stable/Married	1 partner
191	No	Yes	Stable/Married	1 partner
192	No	Yes	Stable/Married	1 partner
193	No	Yes	Stable/Married	1 partner
194	No	Yes	Stable/Married	1 partner
195	No	No	Stable/Married	1 partner
196	Yes	No	Stable/Married	1 partner
197	Yes	Yes	Stable/Married	1 partner
198	No	No	Stable/Married	1 partner
199	No	Yes	Stable/Married	1 partner
200	No	No	Stable/Married	1 partner
201	No	No	Stable/Married	1 partner
202	No	No	Stable/Married	1 partner
203	No	Yes	Stable/Married	1 partner
204	No	Yes	Stable/Married	1 partner
205	Yes	Yes	Stable/Married	1 partner
206	No	Yes	Stable/Married	1 partner
207	Yes	Yes	Stable/Married	1 partner
208	No	No	Stable/Married	2+ partners
209	No	Yes	Stable/Married	1 partner
210	No	No	Stable/Married	1 partner

Subject	HPV	HPV6	HPV11	HPV16	HPV18	HPV26	HPV31	HPV33	HPV35	HPV39	HPV40	HPV42	HPV45	HPV51	HPV52	HPV53	HPV54	HPV55	HPV56	HPV58	HPV59	HPV61	HPV62	HPV66
211	1	0	0	0	0	0	0	0	0	0	0	0	0	0	0	0	0	0	0	0	1	1	0	0
212	1	0	0	0	1	0	0	0	1	0	0	0	0	1	0	0	0	0	0	1	0	0	0	1
213	1	0	0	0	0	0	0	0	0	0	0	0	0	0	0	0	0	0	0	0	0	0	0	0
214	0	0	0	0	0	0	0	0	0	0	0	0	0	0	0	0	0	0	0	0	0	0	0	0
215	0	0	0	0	0	0	0	0	0	0	0	0	0	0	0	0	0	0	0	0	0	0	0	0
216	1	0	0	0	0	0	1	1	0	0	0	1	0	1	0	0	0	0	0	0	0	0	1	0
217	1	0	0	0	0	0	0	0	0	0	0	0	0	0	0	0	1	0	0	0	1	0	0	0
218	1	0	0	0	1	0	1	0	0	0	0	1	0	0	0	0	0	0	0	0	1	0	0	0
219	1	0	0	0	0	0	0	0	0	0	0	0	0	0	0	1	0	0	0	0	0	0	0	0
220	0	0	0	0	0	0	0	0	0	0	0	0	0	0	0	0	0	0	0	0	0	0	0	0
221	0	0	0	0	0	0	0	0	0	0	0	0	0	0	0	0	0	0	0	0	0	0	0	0
222	1	0	0	0	0	0	0	1	1	0	0	0	0	0	0	0	1	1	0	1	0	0	0	1
223	1	0	0	0	0	0	0	0	0	1	0	0	0	0	0	0	0	0	0	0	0	0	0	0
224	1	0	0	0	0	0	0	0	0	0	0	0	1	0	0	0	0	0	0	0	0	0	0	0

Subject	HPV68	HPV69	HPV70	HPV71	HPV72	HPV73	HPV81	HPV82	HPV83	HPV84	HPV89	Age (years)	Age group (years)	Partner has other partners	Living with partner	Site	Injectable contraceptive	Injectable contraceptive type	Condom use
211	0	0	0	0	0	0	0	0	0	0	0	22	18+	Yes	No	Rural	No	None	Yes
212	1	0	0	0	0	0	1	0	0	1	0	22	18+	Don't know	No	Rural	No	None	No
213	0	0	0	0	0	0	0	0	0	1	0	18	18+	Yes	No	Rural	No	None	No
214	0	0	0	0	0	0	0	0	0	0	0	18	18+	No	No	Rural	No	None	No
215	0	0	0	0	0	0	0	0	0	0	0	24	18+	Don't know	No	Rural	Yes	DMPA	Yes
216	0	0	0	0	0	0	0	0	0	0	0	28	18+	Yes	No	Rural	No	None	No
217	0	0	0	0	0	0	0	0	0	0	0	21	18+	No	No	Rural	Yes	DMPA	No
218	0	0	0	0	0	0	0	0	0	1	0	19	18+	Yes	No	Rural	No	None	Yes
219	1	0	0	0	0	0	0	0	0	0	0	23	18+	Yes	No	Rural	Yes	DMPA	No
220	0	0	0	0	0	0	0	0	0	0	0	19	18+	Don't know	No	Rural	No	None	No
221	0	0	0	0	0	0	0	0	0	0	0	20	18+	Don't know	No	Rural	No	None	No
222	0	0	0	0	0	0	0	0	0	0	0	18	18+	No	No	Rural	Yes	DMPA	No
223	0	0	0	1	0	0	0	0	1	0	0	23	18+	Don't know	No	Rural	Yes	DMPA	No
224	0	0	0	0	0	0	0	0	0	0	0	30	18+	Yes	No	Rural	Yes	DMPA	No

Subject	Vaginal insertion practices	Abnormal pelvic exam	Relationship status	Number of sexual partners in the last 3 months
211	No	No	Stable/Married	1 partner
212	No	Yes	Stable/Married	1 partner
213	Yes	No	Stable/Married	1 partner
214	No	No	Stable/Married	1 partner
215	No	Yes	Stable/Married	1 partner
216	No	No	Stable/Married	1 partner
217	No	No	Stable/Married	1 partner
218	No	No	Stable/Married	1 partner
219	Yes	Yes	Stable/Married	1 partner
220	No	No	Stable/Married	1 partner
221	No	Yes	Stable/Married	1 partner
222	No	No	Stable/Married	1 partner
223	No	No	Stable/Married	1 partner
224	Yes	No	Stable/Married	1 partner
