# Supplementary figures and images for: High Burden of Human Papillomavirus (HPV) Infection among Young Women in KwaZulu-Natal, South Africa
Source: PLoS One. 2016 Jan 19;11(1):e0146603. doi: 10.1371/journal.pone.0146603 (PMC4718633; doi:10.1371/journal.pone.0146603)

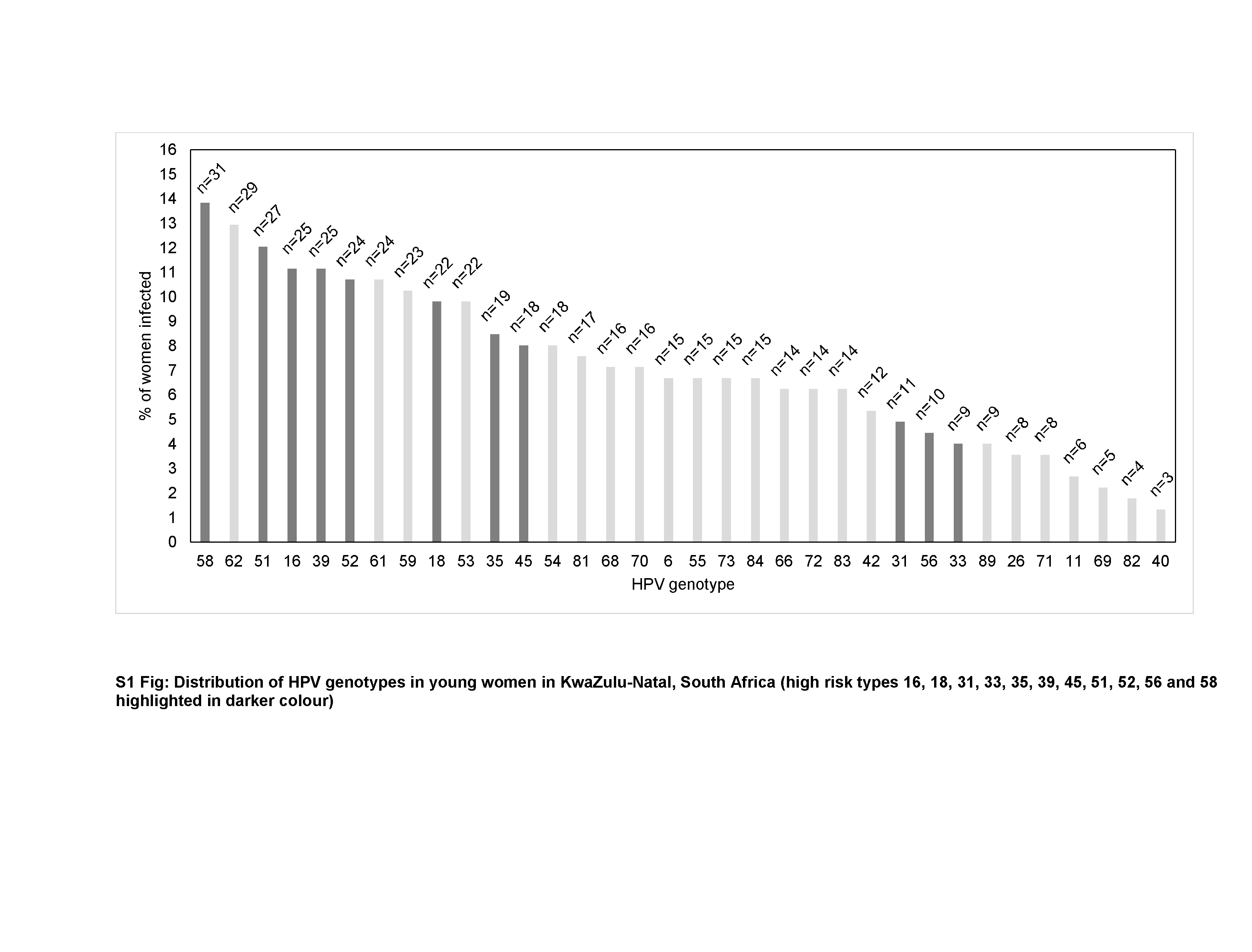

Supplement: S1 Fig — (A) % of women infected. (B) HPV genotype. (TIF) [file pone.0146603.s003.tif]

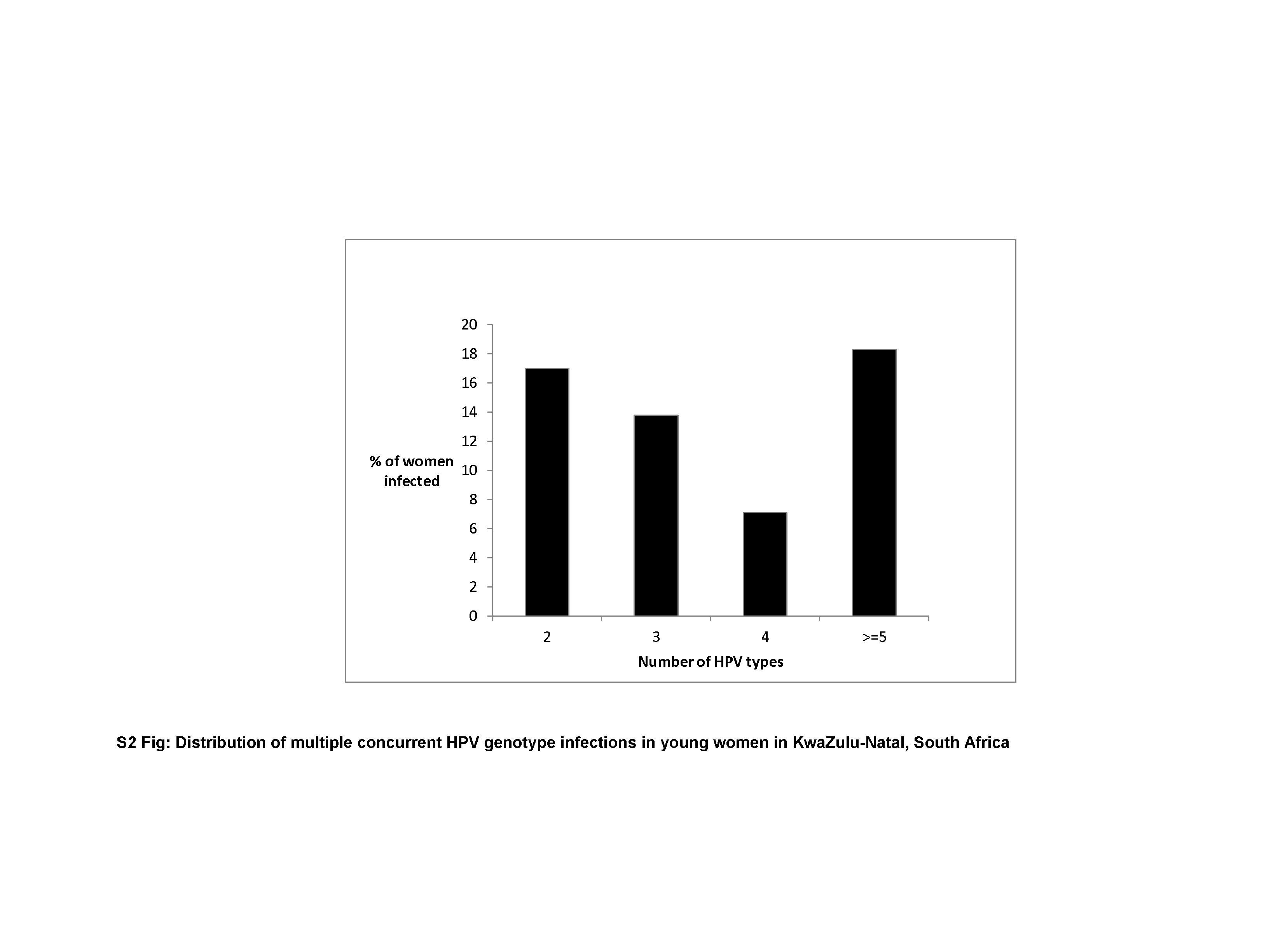

Supplement: S2 Fig — (A) % of women infected. (B) Number of HPV types. (TIF) [file pone.0146603.s004.tif]
